# Statistical analysis and data mining of digital reconstructions of dendritic morphologies

**DOI:** 10.3389/fnana.2014.00138

**Published:** 2014-12-04

**Authors:** Sridevi Polavaram, Todd A. Gillette, Ruchi Parekh, Giorgio A. Ascoli

**Affiliations:** Department of Molecular Neuroscience, Center for Neural Informatics, Structures, and Plasticity, Krasnow Institute for Advanced Study, George Mason UniversityFairfax, VA, USA

**Keywords:** L-Measure (RRID:nif-0000-00003), NeuroMorpho.Org (RRID:nif-0000-00006), neuroinformatics, dendritic topology, cluster analysis, cellular neuroanatomy

## Abstract

Neuronal morphology is diverse among animal species, developmental stages, brain regions, and cell types. The geometry of individual neurons also varies substantially even within the same cell class. Moreover, specific histological, imaging, and reconstruction methodologies can differentially affect morphometric measures. The quantitative characterization of neuronal arbors is necessary for in-depth understanding of the structure-function relationship in nervous systems. The large collection of community-contributed digitally reconstructed neurons available at NeuroMorpho.Org constitutes a “big data” research opportunity for neuroscience discovery beyond the approaches typically pursued in single laboratories. To illustrate these potential and related challenges, we present a database-wide statistical analysis of dendritic arbors enabling the quantification of major morphological similarities and differences across broadly adopted metadata categories. Furthermore, we adopt a complementary unsupervised approach based on clustering and dimensionality reduction to identify the main morphological parameters leading to the most statistically informative structural classification. We find that specific combinations of measures related to branching density, overall size, tortuosity, bifurcation angles, arbor flatness, and topological asymmetry can capture anatomically and functionally relevant features of dendritic trees. The reported results only represent a small fraction of the relationships available for data exploration and hypothesis testing enabled by sharing of digital morphological reconstructions.

## Introduction

**Figure 1:**
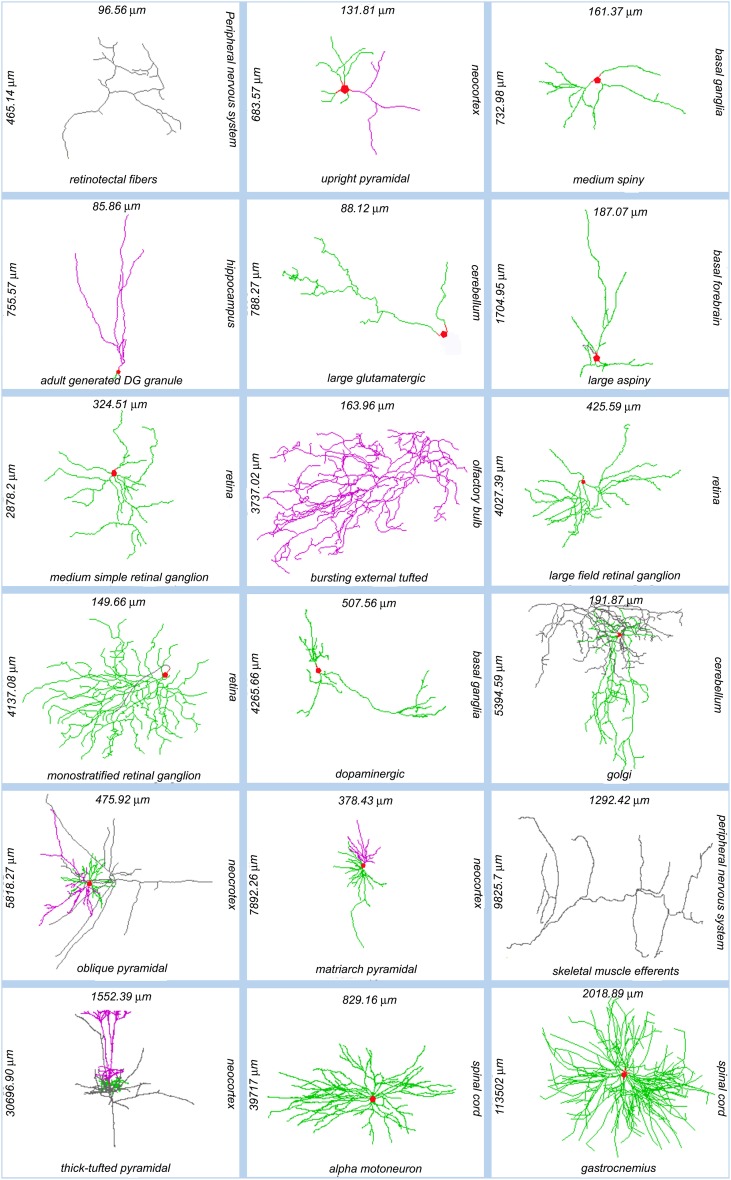
**Sample of NeuroMorpho.Org reconstructions representing the anatomical diversity of dendritic and axonal trees.** Each image is labeled (clockwise from its right side) with the somatic brain region, neuron types, total arbor length, and arbor width. Somata: red; axons: gray; (basal) dendrites: green; apical dendrites: magenta. NeuroMorpho.Org IDs of these neurons from left to right: 06787, 04183, 04457, 06312, 05713, 04477, 00779, 06216, 00777, 05491, 00888, 06904, 06141, 06295, 07707, 07763, 00690, 00606.

The diversity of neuronal morphologies can have broad and profound functional consequences in the nervous system, which have only begun to be understood. Dendritic geometry directly impacts (and mediates) computational processes such as signal integration, coincidence detection, and logical operations (London and Häusser, 2005). The location, orientation, and shape of neural arbors enable (and strongly affect) network connectivity, providing the anatomical substrate to investigate structure-function relationship at the circuitry level (Shepherd and Svoboda, 2005; Briggman and Denk, 2006; Kajiwara et al., 2008; Weiler et al., 2008; Burgalossi et al., 2011; Ropireddy and Ascoli, 2011; Brown et al., 2012). These areas of scientific investigation apply to the morphological differences observed both within and between neuron types across animal species, developmental stages, and brain regions (Figure 1).

**Figure 2:**
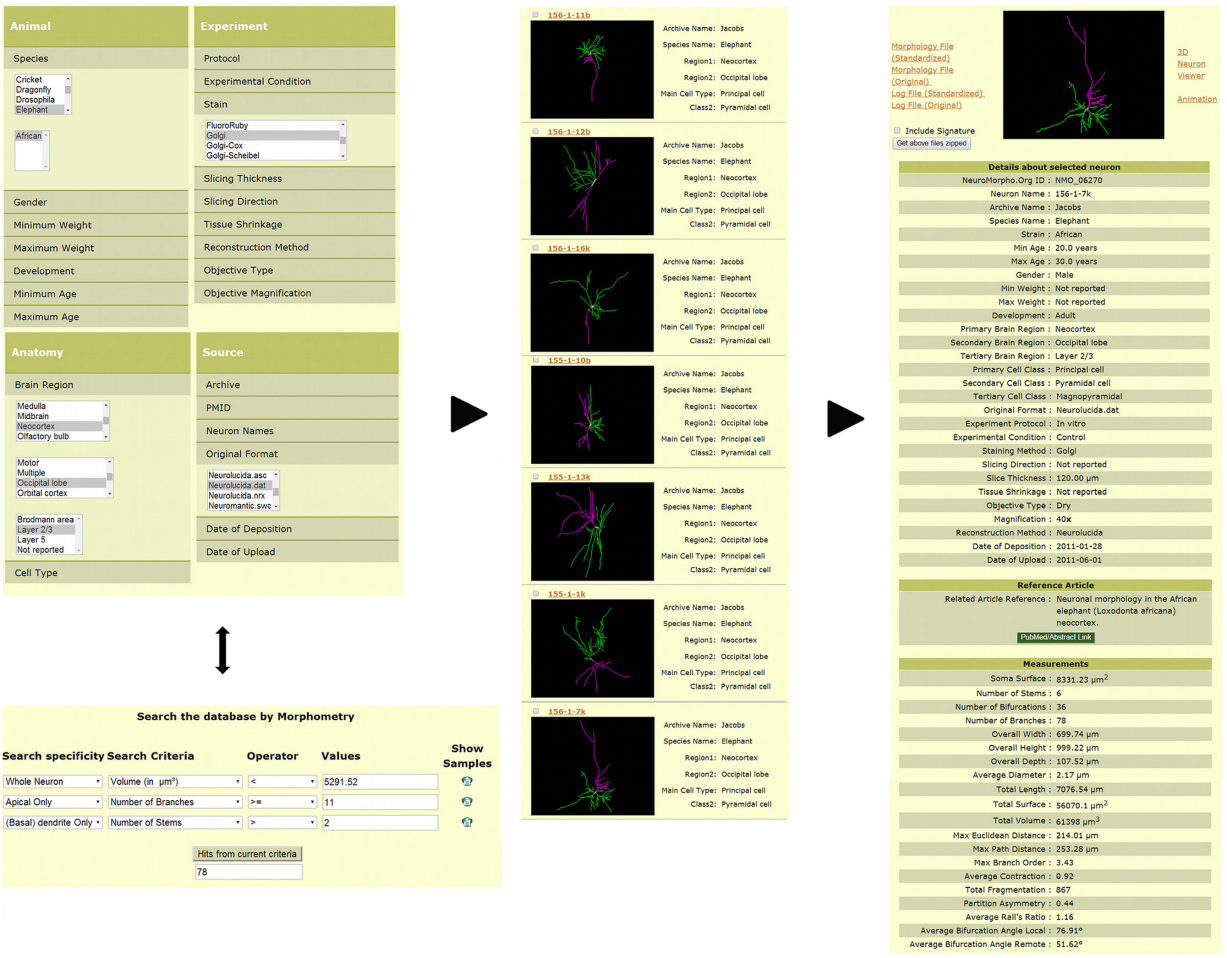
**Search and download features available in NeuroMorpho.Org.** Users can query the database via a number of functionalities to obtain desired reconstructions. The example provided here shows two such options. Reconstructions can be identified by selecting specific metadata across different categories such as species, brain region, cell type, staining method, and original file format (**left panel**, top). Alternatively, reconstructions can be selected by a morphometric search (**left panel**, bottom), wherein users can restrict the search to a specific arbor type (for example, apical dendrites) and define quantitative criteria to restrict particular measures (such as length or number of bifurcations) to ranges of interest. The resulting reconstructions can be displayed (among other options) with a summary of associated metadata **(middle panel)**. The complete metadata and morphometric details are included within each individual neuron page **(right panel)**.

Three-dimensional digital reconstructions of axonal and dendritic arbors, combined with neuroinformatics tools and computational approaches, allow considerable opportunities for data processing, analysis, and modeling at both cellular- and systems-level (Parekh and Ascoli, 2013). The open availability of these reconstructions in databases such as NeuroMorpho.Org (Figure 2) enables re-analysis of shared data (Ascoli, 2007). As of version 5.6, the repository contained over 10,000 reconstructions contributed by 120 laboratories from 21 species, 85 brain regions and 123 cell types, representing more than 240,000 hours of manual tracing. NeuroMorpho.Org users can browse the data by animal species, brain region, cell type, and contributing lab. The “search by” option can be used to select and combine specific metadata criteria (Figure 2, left panel top) from a drop-down menu of categories such as developmental stage, experimental condition, and reconstruction method. The morphometry search functionality (Figure 2, left panel bottom) allows users to find neurons matching any combination of more than 20 morphometric criteria. From the resulting summary list of neurons (Figure 2, middle panel), individual pages for each reconstruction can be retrieved, thus displaying related metadata, a link to the associated publication, and the pre-computed morphometrics (Figure 2, right panel). Each reconstruction is downloadable as the standardized version along with the original contributed version. The log files detailing the changes made during the standardization process are available for download as well. From the individual neuron pages, users can also launch an animation file and an interactive 3D viewer.

Quantitative morphometry of neuronal reconstructions is often used for shape analysis (Uylings and van Pelt, 2002; Van Ooyen et al., 2002; Rocchi et al., 2007), also in conjunction with biologically-inspired computational simulations (Ascoli et al., 2001; Van Ooyen, 2011). For example, statistical distribution of morphological features are used in stochastic growth algorithms for generating virtual trees (Van Pelt et al., 1997; Donohue and Ascoli, 2008; Koene et al., 2009; Evans and Polavaram, 2013; Memelli et al., 2013). Moreover, statistical analyses of neuronal reconstructions facilitate and support theoretical investigations. These studies for instance provided evidence for optimal wiring principles of neuronal arbors (Wen and Chklovskii, 2008) and their power law distributions, which may relate to synaptic input sampling (Lee and Stevens, 2007; Snider et al., 2010; Teeter and Stevens, 2011; Cuntz et al., 2012).

This study uses the L-Measure software tool (Scorcioni et al., 2008) to extract morphometric data from neuronal arbors for large scale statistical analyses of available data. L-Measure computes simple statistics of morphometric features as well as their frequency distribution and inter-dependence (e.g., how arbor length varies with path distance from the soma). This tool has been used in a broad range of applications, including multidimensional analysis of neuronal shape (Costa et al., 2010; Zawadzki et al., 2012) and comparative studies of sensory neurons in the fly (Ting et al., 2014) and of respiratory neurons in the pre-Bötzinger complex (Koizumi et al., 2013). In conjunction with L-Neuron (Ascoli and Krichmar, 2000), L-Measure has also been employed to generate and validate a large-scale model of the dentate gyrus with half a million neurons (Schneider et al., 2012). L-Measure has also enabled analysis of non-neuronal arbors such as arterial vasculature (Wright et al., 2013), and was integrated into other digital reconstruction and analysis systems, such as the Farsight toolkit (http://farsight-toolkit.org) and Vaa3D (https://code.google.com/p/vaa3d).

With the first successes in high-throughput automatic digital neuronal tracing (Chiang et al., 2011) and overall increasing volumes of published and shared reconstructions (Halavi et al., 2012), “big data” opportunities for knowledge mining are starting to emerge. On the one hand, this increasing availability of shared data may foster remarkable discoveries. On the other, the heterogeneous source of data and disparate experimental conditions also pose non-trivial challenges to database-wide analyses. As a step toward large database analysis, here we utilize exploratory data analysis to quantify morphological similarities and differences across broadly diverse dendritic arbors. In the process, we recognize several critical limitations when pooling together widely non-uniform data sets. Consequently, we propose selection criteria and methodological choices aimed to maximize the potential biological relevance of the results. With such a research design, dimensionality reduction and unsupervised clustering reveal tentative morphological relationships between specific neuron types involving branching density, topology, size, and tortuosity. At the same time, we identify the most delicate factors in both data and metadata that must be considered to optimize the impact of future large-scale morphological investigations.

## Methods

### Selection of datasets and morphometric features for analysis

The entire pool of 10,004 reconstructions downloaded from NeuroMorpho.Org v5.6 was screened for a pre-determined set of inclusion criteria to improve interpretability of the results. Specifically, in order to be considered for analysis, digital neuron reconstructions had to (a) belong to the “control” experimental condition; (b) contain at least four dendritic bifurcations; (c) include branch-path information and not just bifurcation connectivity; and (d) have non-zero depth range (eliminating two-dimensional tracings). The 7,143 reconstructions matching these characteristics were analyzed by their NeuroMorpho.Org metadata assignments to specific animal species, brain region, and cell type. Subsequently, for the purpose of cluster analysis chi-square testing (see below), groups of fewer than 40 neurons in any metadata combination of species, brain region, cell type, and lab of origin were excluded to ensure sufficient statistical power (Yates et al., 1999). This further selection reduced the number of reconstructions to 5,099, divided into 45 unique metadata groups.

Because of the major differences between axonal and dendritic morphology, and the remarkable abundance of reconstructed dendrites relative to axons, only dendritic arbors were included in this study. Focusing on a more consistent and comparable dataset allows addressing more biologically relevant questions. Moreover, this choice reduces the errors due to incomplete reconstructions, which are considerably more severe for projection axons than for dendrites.

L-Measure allows extraction of approximately 100 distinct features from each neuron (see http://cng.gmu.edu:8080/Lm for full listing and detailed definitions). Of these, all measures related to branch diameter were excluded due to their strong dependence on imaging resolution, optical magnification, and other experimental details causing excessive inter-laboratory variability (Scorcioni et al., 2004). All other features were subjected to cross-correlation analysis, and those with correlation greater than 80% were sequentially eliminated one at a time (re-running the cross-correlation at each step) as they were considered highly redundant with the rest of the features. This selection left 27 features (Table 1) that were used for the remainder of the analysis. Dendritic arbor size measures consisted of total length, number of tips, height, width, and depth. Bifurcation measures included average partition asymmetry as well as amplitude, tilt, and torque angles measured locally with the adjacent tracing points or remotely with the preceding and following bifurcations or terminations. Branch measures consisted of length, tortuosity, and fractal dimension. Lastly, local measures included branch order, terminal degree, path distance from soma, and helicity.

**Table 1:** **Coefficients of variation of all L-Measure derived morphometric features**.

**Morphometric features**	**CV for Dendrites**	
	**Hierarchy groups**	**Cluster groups**
**I. WHOLE TREE/NEURON SIZE**		
Summed total arbor length	1.38	0.57
Number of arbor tips	1.65	1.82
Total arbor width	0.68	0.43
Total arbor height	0.65	0.51
Total arbor depth	1.12	0.65
**II. BIFURCATION MEASURES**		
Avg. partition asymmetry	0.27	0.26
Avg. local amplitude angle	0.17	0.17
Max. local amplitude angle	0.19	0.18
Avg. remote amplitude angle	0.21	0.18
Max. remote amplitude angle	0.24	0.23
Avg. local tilt angle	0.14	0.13
Max. local tilt angle	0.08	0.08
Avg. remote tilt angle	0.09	0.08
Max. remote tilt angle	0.05	0.05
Avg. local torque angle	0.17	0.16
Max. local torque angle	0.11	0.11
Avg. remote torque angle	0.18	0.17
Max. remote torque angle	0.10	0.10
**III. BRANCH MEASURES**		
Avg. tortuosity	0.08	0.07
Avg. fractal dimension	0.03	0.02
Max. fractal dimension	0.15	0.14
Avg. branch path length	0.59	0.41
Max. branch path length	0.81	0.53
**IV. COMPARTMENT MEASURES**		
Max. branch order	0.85	0.85
Avg. terminal degree	0.71	0.68
Max. path distance from soma	0.76	0.57
Max. branch helicity	0.19	0.16

A detailed description of each metric is provided at http://cng.gmu.edu:8080/Lm/help/index.htm.

### Principal component analysis (PCA) and cluster analysis

In order to reduce the dimensionality of the morphometric space for unsupervised clustering, PCA was run on the feature dataset using the “*prcomp*” routine in R (v. 2.15.1). This transformation rotates all extracted measures (27 features for 5,099 arbors) such that the first dimensions in the new space capture the most variance (in decreasing order). Prior to PCA, all features were normalized by their respective standard deviations, and the features with absolute skewness greater than unity (17/27) were log-transformed. Negatively skewed distributions were inverted and distributions with negative values were shifted prior to log-transformation. These steps ensure an approximately normal distribution of the input features, as assumed by PCA and subsequent clustering. The resulting first 17 components, accounting for 95% of the variance, were retained for cluster analysis.

Next, the dendritic arbors were clustered based on their principal morphometric components to seek a shape-based classification independent of *a priori* metadata grouping. We selected a model-based approach, in which mixtures of Gaussians are found that together have maximal likelihood to fit the data. A cluster is the collection of arbors that are most likely to come from the same multivariate Gaussian (referred to as a cluster model). We used the R “*MCLUST*” package (Farley and Raftery, 2006) for estimating optimal model parameters and selecting the most likely model type given the dataset. The model types include spherical, ellipsoidal (with a diagonal covariance matrix), and ellipsoidal with orientation (indicating correlation between dimensions). This flexibility makes model-based clustering a more suitable choice than other popular methods (e.g., K-means) for analysis of heterogeneous data sets collated from different experiments, labs, and conditions. Not only are clusters not limited to fit spherically symmetric distributions, but also each cluster is allowed to have its own distinct variance, shape, and orientation.

MCLUST implements Expectation Maximization (EM) to select models using the Bayesian information criterion (BIC). The BIC computes the log likelihood of the cluster model, but includes a penalty for the number of parameters weighted by the log of the dataset size. Thus, goodness of fit is balanced against model simplicity according to the following equation, whereby the largest value determines the best model:

(1) $$\text{BIC}=-2\cdot \ln\hat{L}+k\cdot \ln(n)$$

Here, $\hat{L}$ is the maximized likelihood computed on the marginal likelihood P(y|M_i_) of the candidate model M_i_ given the observed data y (y_1_, … y_n_); *k* is the number of free parameters to be estimated; and *n* is the number of data points.

The specification of MCLUST model types and parameters is coded by three letters in each of three positions. The three positions represent the model size, shape, and orientation variables, respectively. Letter “E” indicates that the parameters are equivalent across all clusters, “V” signifies variable parameter values, and “I” denotes that the corresponding parameter is not applicable. For example, “EII” indicates spherical Gaussians (no shape or orientation) with equal size among clusters, which corresponds to the traditional K-means method. Similarly, the “VVV” model type indicates varying size, shape, and orientation parameters. This latter model was determined by EM to be optimal for the data analyzed here despite its greater BIC cost implied by the larger number of free parameters. Thus, EM provides information theory-derived evidence that the performance of simple uniform spherical (K-means-like) clustering is sub-optimal for the data used in this study.

Cluster distances from the center of coordinates were measured by Z score to account for relative variance. Pairwise cluster distances were computed as the distances between the corresponding centers normalized by the cluster scatters, which are defined as averaged distance of the cluster points from the respective cluster center (Dunn, 1973). The associations among clusters and metadata groups were assessed using the chi-square test of independence, using the (marginal) frequencies of group and cluster occurrences to calculate the expected association matrix, and computing the Bonferroni-corrected *p*-values of the observed co-occurrences from the standardized residuals.

## Results

### Variability of dendritic morphology and comparison by metadata

To quantify the heterogeneity of the data, we computed the coefficient of variation (CV) for each of the 27 measured features over the entire set of 7,143 neurons as well as over the subset of 5,099 neurons used in cluster analysis (Table 1). Tortuosity, fractal dimension, and tilt angle are the least variable features, with a CV of less than 10%. In contrast, size measures are the most variable, with a CV close to or greater than unity. This apparent distinction between “local” (branch-level) vs. “global” (neuron-level) features may reflect both the effect of biological constraints (e.g., varying dimensions of different species from insects to human) and experimental conditions (slice vs. whole-animal preparations). Most other metrics display intermediate CV values.

**Figure 3:**
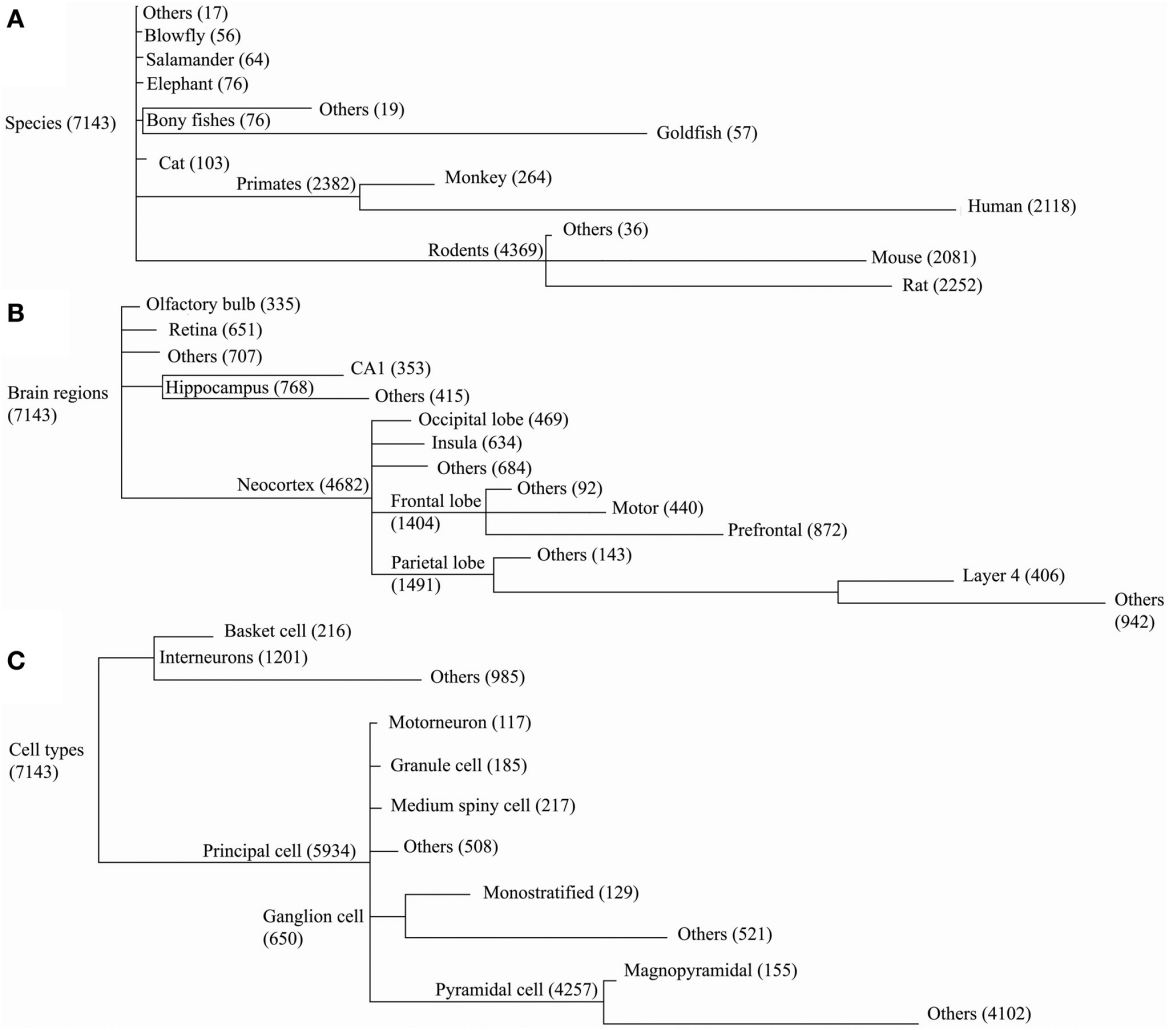
**NeuroMorpho.Org v5.6 data is categorized along three major metadata dimensions, namely species, brain regions, and cell types.** Reconstructions are hierarchically organized in each of these dimensions. Every node in the hierarchy is labeled by the number of associated reconstructions. The line lengths are proportional to the size of the child nodes relative to their parent nodes. **(A)** In the species hierarchy nodes with fewer than 55 reconstructions are grouped together with the “not reported” data under “Others.” In the brain regions **(B)** and cell type **(C)** hierarchies the grouping thresholds are 300 and 100, respectively.

Dendritic morphologies were then compared across species, cell types, and brain regions. The corresponding metadata information for each reconstruction in NeuroMorpho.Org was organized hierarchically (Figure 3), forming groups with a sufficient number of neurons to enable statistical comparison of the results (at least 55 for species, 300 for brain regions, and 100 for cell types). Groups with fewer reconstructions were combined into “others” together with the reconstructions missing the detailed metadata information at the corresponding level of the hierarchy (marked as “not reported” in NeuroMorpho.Org).

**Figure 4:**
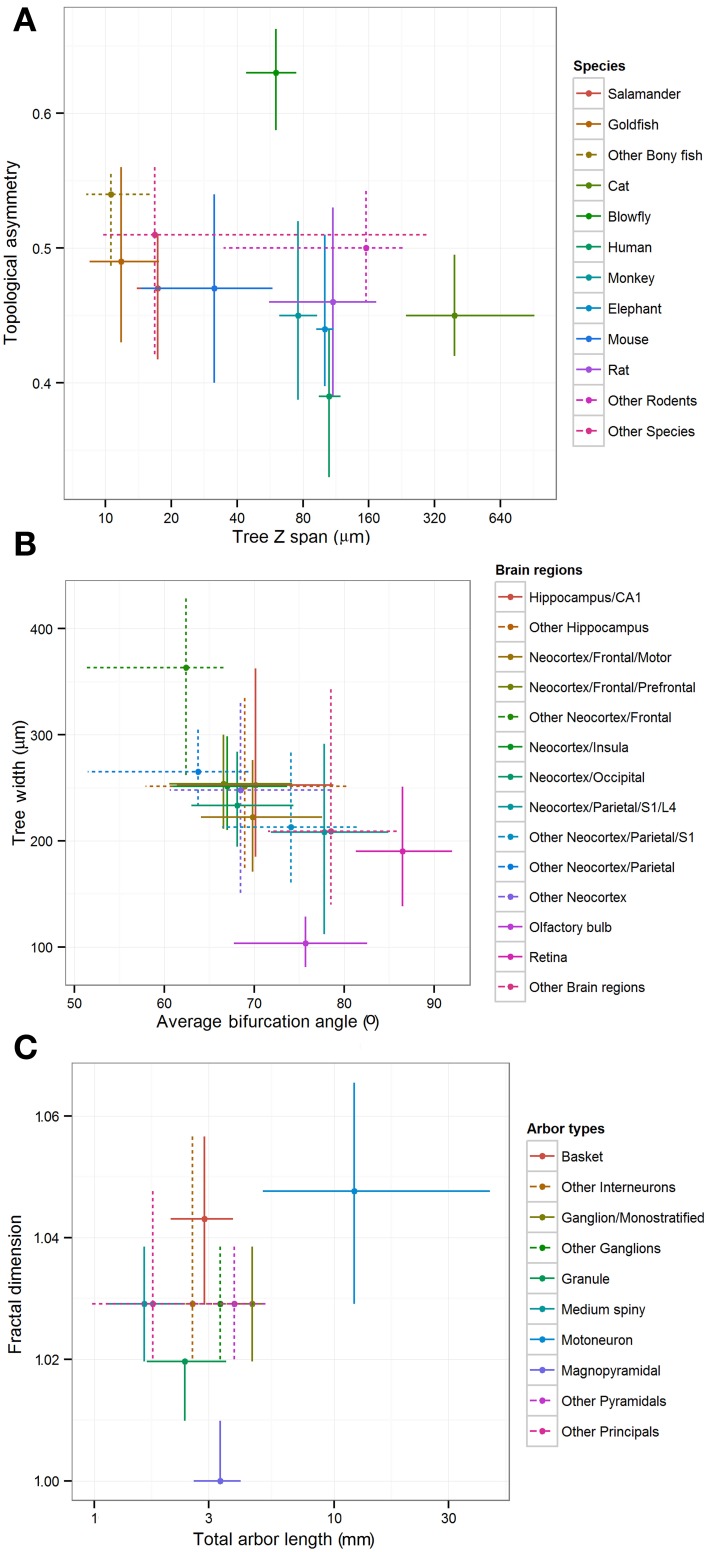
**Inter-group differences of representative morphometric features within each main metadata dimension.** Crosshairs represent medians and quartile ranges of each group corresponding to the leaf nodes in the hierarchies shown in Figure 3. Dotted lines indicate “other” groups with merged data. **(A)** Differences in arbor depth and topological asymmetry among species. **(B)** Differences in arbor width and average bifurcation angle among brain regions. **(C)** Differences in fractal dimension and total arbor length among cell types.

The “leaf” nodes in each of the three metadata hierarchies (12 for species, 14 for brain regions, and 10 for cell types) were compared with a selection of representative morphometric features (Figure 4). In a limited set of cases, individual groups could be distinguished from the rest or from each other. For example, blowfly and cat reconstructions stood out against the neurons of all other species for their large topological asymmetry and Z span, respectively. The dendritic arbors of magnopyramidal cells tended to have extensive total length but low fractal dimension, whereas granule cells displayed opposite characteristics. At the same time, most groups show extensive overlap of their morphometric variance, preventing firm statistical conclusions. Part of the reason for such broad distributions is likely due to the non-uniform nature of archive-wide data sets pooled together across different experiments and laboratories. It is also clear that these metadata dimensions are not mutually independent because of evolutionary constraints (e.g., bony fishes lack a neocortex) and the finite sample of reconstructions (e.g., all monostratified ganglion cells came from the mouse retina). More generally, while popular in comparative anatomy, such a pairwise approach lacks the ability to reveal multivariate effects that are unavoidable given the non-random association between metadata groups and experimental conditions.

### Extracting primary morphological features by PCA and cluster models

In order to overcome the above limitations, we adopted an unsupervised clustering approach following dimensionality reduction with PCA. The first step is to reduce the initial parameter space to fewer orthogonal dimensions capturing most of the data variability. In mathematical terms, PCA identifies the linearly independent combinations of variables ordered by the amount of variance they explain. From the (27) original morphometric features, the first 17 dimensions of PCA covered 95% of the data variance and were used for cluster analysis.

The first 6 of these principal components were responsible for three quarters of the variance and displayed distinctive compositions of their primary morphometric features (Table 2). Identifying the heaviest contributors in the linear combination of morphometric features of each principal component (“loadings”) is useful to aid subsequent interpretation of the results. The first component (PC1) is positively loaded on bifurcation angles and negatively on branch path length, reflecting high branching density. The morphometric features most descriptive of PC2 and PC3 are respectively overall size and branch tortuosity. Together, the first three components capture the majority of the data variance. The simplest morphological descriptors of PC4, PC5, and PC6 are arbor flatness (related to torque angle), fractal dimension (or “space filling”), and topological asymmetry (the average normalized sub-tree partition at bifurcation points), respectively.

**Table 2:** **Primary morphometric loading (with absolute values of 0.25 or higher) of the first six principal components of the dendritic arbors used in cluster analysis**.

**Principal Component**	**Morphometric features**	**Loading**
PC1 (27% of cumulative variance): branching density	Max. remote amplitude angle	0.29
	Avg. remote amplitude angle	0.27
	Max. local amplitude angle	0.26
	Avg. terminal degree	0.25
	Max. branch order	0.25
	Avg. branch path length	−0.28
	Avg. remote tilt angle	−0.26
PC2 (43% of cumulative variance): size	Summed total arbor length	0.4
	Total arbor height	0.36
	Max. path distance from soma	0.34
	Total arbor width	0.33
PC3 (58% of cumulative variance): branch tortuosity	Avg. tortuosity	0.42
	Avg. fractal dimension	0.34
	Avg. local tilt angle	−0.34
PC4 (64% of cumulative variance): arbor flatness	Avg. remote torque angle	0.63
	Avg. local torque angle	0.62
PC5 (70% of cumulative variance): fractal dimension and tilt angles	Max. fractal dimension	0.37
	Avg. fractal dimension	0.35
	Avg. remote tilt angle	0.35
	Avg. tortuosity	0.25
	Max. remote tilt angle	−0.32
	Avg. remote amplitude angle	−0.36
PC6 (75% of cumulative variance): partition asymmetry and depth	Avg. partition asymmetry	0.41
	Total arbor depth	0.35

**Figure 5:**
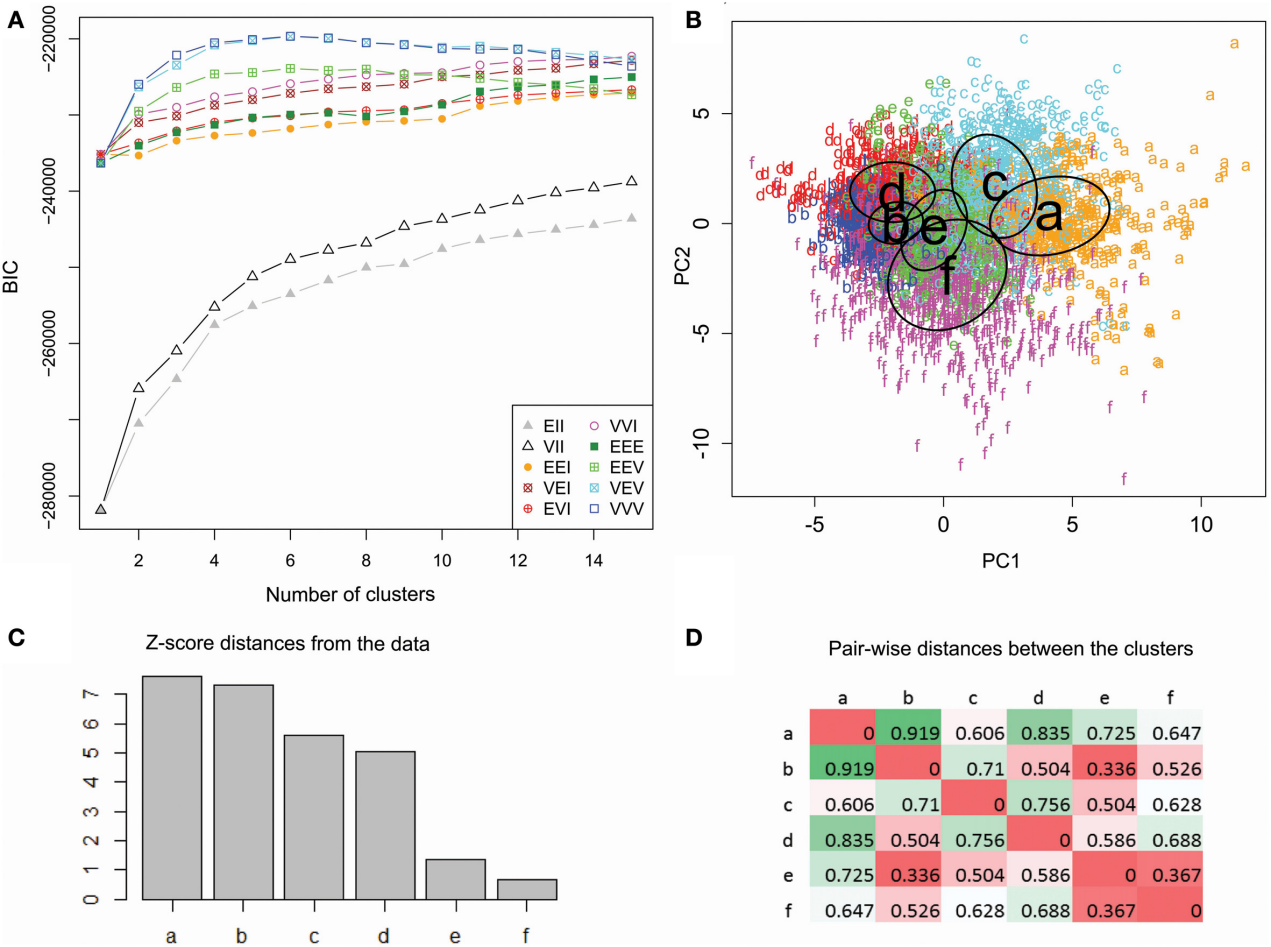
**Unsupervised cluster analysis of dendritic morphology. (A)** Maximization of BIC reveals marginal performance of spherical clustering with equal (EII) or unequal size (VII) alike relative to the models allowing ellipsoidal clusters. Among those, those accounting for unequal orientation (EEV, VEV, and VVV) performed better, especially with unequal size (VEV and VVV). The highest BIC value was attained at 6 clusters with varying size, shapes and orientation (VVV). **(B)** Scatter plot of color-coded cluster assignments (*a* through *f*) projected on the first two principal components. The ovals represent best fitting cluster parameters. **(C)** Cluster ranking by Z score distance from the origin of coordinates. **(D)** Pairwise inter-cluster distances normalized by the corresponding scatters. Farthest distances are in green and nearest are in red.

In order to produce the most informative statistical model, unsupervised clustering selects the optimal number of clusters as well as their parameters, by maximizing the BIC. These data were best fit to six clusters with varying size, shape, and orientation (Figure 5). The numerical difference between this model and the variant with constant cluster shape, however, was minimal (and is undetectable in Figure 5A). The same model type, moreover, performed nearly as well with five or seven clusters as indicated by the absence of a clear peak in the BIC plot. We experimented with these alternative model variant and numbers of clusters and found no substantial differences in findings. At the same time, the data were *not* adequately described by traditional spherical clusters, even if with unequal sizes (Figure 5A).

Since six clusters correspond to the maximum value for both top model types, we selected this number as the most suitable for exploratory analysis. Such a choice, nevertheless, should not be taken to reflect a ground truth that only six “true” classes exist within the data. This selection simply maximizes the inter-similarity of co-clustered classes relative to classes in other clusters given the scope, size, quality, and composition of the available dataset. To determine if further differences exist between classes that associate with the same cluster, it would be appropriate to run the same analysis on a subset of the data (sub-clustering). This additional analysis, however, requires larger datasets to meet the selection criteria based on a minimum number of reconstructions in each dataset.

The two-dimensional data projection on the first and second components illustrates the relative discrimination of clusters by branching density and arbor size (Figure 5B). Cluster ranking by variance-normalized distance from the center of coordinates (Figure 5C) allows for focused analysis on clusters farther from the origin (*a–d*), and thus morphologically distinctive, relative to those closer (*e* and *f*) to the origin. The six clusters contain respectively 585 (*a*), 1488 (*b*), 762 (*c*), 555 (*d*), 818 (*e*), and 891 (*f*) reconstructions. Pairwise distances (Figure 5D) reveal that one and the same cluster (*b*) is both the farthest from (*a*) and closest to (*e*) than to other clusters.

### Statistical associations between clusters and metadata combinations

Unsupervised cluster models segregate neuronal reconstructions solely based on morphological features. This classification is thus complementary to, and independent of, the metadata associated with each reconstruction. The correspondence between the six morphological clusters and the 45 unique metadata groups characterized by species, brain region, neuron type, and lab of origin can shed light on the most important morphometric signatures of each metadata group. The 45-by-6 chi-square contingency matrix (Table 3) reports the probabilities that the observed over-representation and under-representations of associations between morphological clusters and metadata groups would be due to chance if the observed numerical compositions of each cluster and group were independent of each other. For example (first data row in Table 3), pyramidal neurons from mouse primary somatosensory cortex in Smit–Rigter's archive are significantly over-represented in cluster *a* (*p* < 0.0002 = 10^−3.73^) and significantly under-represented in cluster *b* (*p* < 0.001 = 10^−3.05^). In contrast, the proportion of these same neurons in cluster *d* is within the range expected from the sizes of this metadata group and morphological cluster.

**Table 3:**
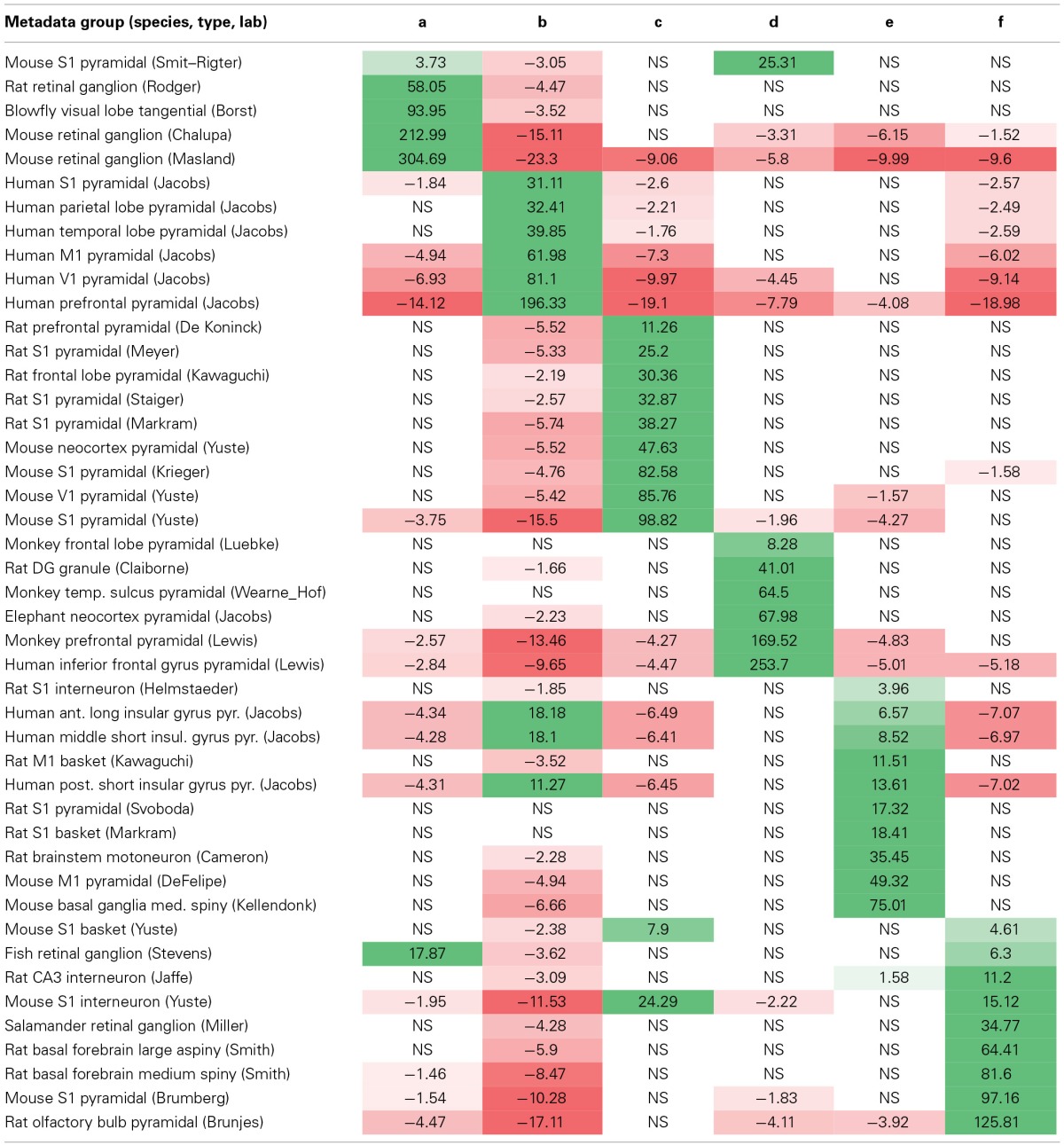
**Matrix of positive (green) and negative (red) associations between metadata groups (rows) and morphological clusters (columns)**.

The Bonferroni adjusted p-values obtained by the chi-square test of independence are converted for ease of comparison into log_10_ values, inverting the sign for overrepresented (green) cells. The color gradient shows the interaction strength. Non-significant (p > 0.05) associations are indicated with NS.

Interestingly, each and every metadata group is over-represented in, and thus associated with, one of the six morphological clusters. The majority (38/45) are associated with exactly one cluster, and all of the remaining (7/45) are each split between just two clusters. Most possible metadata/cluster pairs deviated significantly from the random distribution expected from the “null hypothesis”: 53 out of 270 were significantly over-represented and 87 out of 270 significantly under-represented. This overall partition of metadata groups in distinct clusters constitutes a remarkable outcome for a fully unsupervised classification method. Certain metadata groups are over-represented in one morphological cluster and under-represented in all other clusters, such as ganglion cells from mouse retina in Masland's archive (cluster *a*) and pyramidal cells from human prefrontal cortex in Jacobs' archive (cluster *b*). Other metadata groups are over-represented in one morphological cluster, but otherwise scattered throughout all other clusters per the respective numerical abundance, such as pyramidal cells from monkey frontal lobe in Luebke's archive (cluster *d*) and motoneurons from rat brainstem in Cameron's archive (cluster *e)*.

Several observations can be made that transcend individual archive identities. All rodent retinal ganglion cell groups are associated with cluster *a*, whereas fish and salamander retinal ganglion cells groups are associated with cluster *f*. The relative cluster positions in the first two principal components and the corresponding morphological loadings (Figure 5B and Table 2) suggest that the retinal ganglion cells are larger and with denser branching in rodents than in non-mammals. Neocortex pyramidal cell groups are distributed across all clusters, with preference mostly based on species (most notably, human in *b*, rodents in *c*, and monkey in *d*). All rodent non-cortical and non-pyramidal cell groups are found in cluster *f* (along with salamander and fish retinal ganglion cells). Such metadata heterogeneity, together with this cluster's minimal distance from the morphological center (Figure 5C) suggests a putative “catch-all” role for cluster *f*, which makes it broadly representative of the whole dataset.

In several cases, the split of a metadata group into two morphological clusters reflects previously reported relations. For example, three groups of pyramidal neurons from the (anterior, middle, and posterior) human insular gyrus in the Jacobs' archive divided between clusters *b* and *e* according to structural differences related to the subject's gender (Anderson et al., 2009). Similarly, mouse primary somatosensory pyramidal cells are over-represented in both clusters *a* and *d*, consistent with the reported differences between young and adult animals (Smit-Rigter et al., 2012). The grouping of neurons from younger mice with retinal ganglion cells (in cluster *a*) and from the older mice with pyramidal cells of larger mammals, such as monkey, elephant, and human (in cluster *d*), could be expected since the former groups are characterized by the shortest branch path length and the latter groups by the largest. The scattered clustering of pyramidal neurons, however, does not necessarily reflect existing biological relations, but might rather result from the combination of the choice of analysis algorithms, selection of parameters, and experimental differences.

The other splits of metadata groups between two clusters (Table 3) similarly revealed differences likely due to experimental procedures, such as staining protocol or slicing direction, which were not recognized in the original reports (Anderson et al., 1995; Soloway et al., 2002; Goldberg et al., 2003; MacLean et al., 2005; Nikolenko et al., 2007; Woodruff et al., 2009). For example, the separate clustering of different mouse S1 pyramidal cell datasets can be explained by the differences between intracellular biocytin injection (e.g., Yuste's archive) and bulk Golgi staining (e.g., Brumberg's archive). While the mechanisms underlying the different visualization by these techniques are not yet fully understood (Thomson and Armstrong, 2011), the histological labeling information is available as metadata in NeuroMorpho.Org, thus aiding interpretation.

A complementary way to examine the associations between morphological clusters and metadata groups is to systematically analyze the composition of each cluster in terms of its associated groups, broken down by fraction of group, fraction of cluster, and neuron count (Table 4). For example (first data row in Table 4), 33% of the mouse S1 pyramidal cells from the Smit–Rigter archive are in cluster *a*, accounting for only 3% of this cluster (17 out of 560 neurons). The sums of cluster fractions in Table 4 correspond to the proportion of neurons in each cluster (e.g., 97% for cluster *a*) made up by the cluster's associated metadata groups (green entries in Table 3). The remaining portions of the clusters are composed of neurons falling outside of their associated cluster. Notably, the blowfly tangential cell group is associated with cluster *a*. Moreover, clusters *b* and *c* are exclusively associated with human pyramidal cell (in which only basal dendrites are reconstructed) and rodent neocortex cell groups respectively.

**Table 4:** **Composition of the six morphological clusters in terms of their over-represented metadata groups**.

**Cluster**	**Metadata group**	**Fraction of group**	**Fraction of cluster**	**Counts**
**a**	Mouse S1 pyramidal (Smit–Rigter)	0.33	0.03	17
	Fish retinal ganglion (Stevens)	0.51	0.05	29
	Rat retinal ganglion (Rodger)	0.76	0.09	50
	Mouse retinal ganglion (Chalupa)	0.85	0.26	151
	Mouse retinal ganglion (Masland)	0.99	0.44	257
	Blowfly visual lobe tangential (Borst)	1	0.1	56
	**Total**		**0.97**	**560**
**b**	Human posterior short insular gyrus pyramidal (Jacobs)	0.53	0.07	106
	Human anterior long insular gyrus pyramidal (Jacobs)	0.59	0.08	118
	Human middle short insular gyrus pyramidal (Jacobs)	0.59	0.08	117
	Human S1 pyramidal (Jacobs)	0.79	0.06	95
	Human V1 pyramidal (Jacobs)	0.8	0.15	226
	Human M1 pyramidal (Jacobs)	0.8	0.12	176
	Human parietal lobe pyramidal (Jacobs)	0.86	0.06	84
	Human prefrontal pyramidal (Jacobs)	0.88	0.29	434
	Human temporal lobe pyramidal (Jacobs)	0.91	0.06	91
	**Total**		**0.97**	**1447**
**c**	Rat prefrontal pyramidal (De Koninck)	0.43	0.05	39
	Mouse S1 interneuron (Yuste)	0.47	0.09	66
	Mouse S1 basket (Yuste)	0.5	0.03	22
	Rat S1 pyramidal (Meyer)	0.6	0.06	45
	Rat S1 pyramidal (Markram)	0.66	0.07	57
	Mouse S1 pyramidal (Yuste)	0.71	0.17	128
	Mouse neocortex pyramidal (Yuste)	0.75	0.08	58
	Rat S1 pyramidal (Staiger)	0.8	0.05	37
	Rat frontal lobe pyramidal (Kawaguchi)	0.81	0.04	34
	Mouse V1 pyramidal (Yuste)	0.96	0.1	73
	Mouse S1 pyramidal (Krieger)	0.99	0.09	68
	**Total**		**0.83**	**627**
**d**	Monkey frontal lobe pyramidal (Luebke)	0.43	0.03	18
	Monkey S1 pyramidal (Smit–Rigter)	0.59	0.05	30
	Rat DG granule (Claiborne)	0.77	0.06	33
	Monkey prefrontal pyramidal (Lewis)	0.79	0.23	126
	Elephant neocortex pyramidal (Jacobs)	0.9	0.08	44
	Monkey temporal sulcus pyramidal (Wearne_Hof)	0.93	0.07	40
	Human inferior frontal gyrus pyramidal (Lewis)	0.96	0.26	146
	**Total**		**0.78**	**437**
**e**	Human anterior long insular gyrus pyramidal (Jacobs)	0.32	0.08	63
	Human middle short insular gyrus pyramidal (Jacobs)	0.33	0.08	66
	Rat CA3 interneuron (Jaffe)	0.34	0.02	20
	Human posterior short insular gyrus pyramidal (Jacobs)	0.37	0.09	74
	Rat S1 interneuron (Helmstaeder)	0.4	0.03	23
	Rat M1 basket (Kawaguchi)	0.54	0.04	30
	Rat S1 pyramidal (Svoboda)	0.58	0.05	38
	Rat S1 basket (Markram)	0.65	0.04	33
	Mouse M1 pyramidal (DeFelipe)	0.74	0.08	67
	Mouse basal ganglia medium spiny (Kellendonk)	0.83	0.1	85
	Rat brainstem motoneuron (Cameron)	0.88	0.05	38
	**Total**		**0.66**	**537**
**f**	Mouse S1 interneuron (Yuste)	0.45	0.07	63
	Fish retinal ganglion (Stevens)	0.47	0.03	27
	Mouse S1 basket (Yuste)	0.48	0.02	21
	Rat CA3 interneuron (Jaffe)	0.55	0.04	32
	Salamander retinal ganglion (Miller)	0.78	0.06	50
	Rat olfactory bulb pyramidal (Brunjes)	0.8	0.18	164
	Mouse S1 pyramidal (Brumberg)	0.88	0.13	112
	Rat basal forebrain medium spiny (Smith)	0.88	0.11	95
	Rat basal forebrain large aspiny (Smith)	0.9	0.08	73
	**Total**		**0.72**	**637**

Associations between metadata groups and morphological clusters are quantified as fraction of the group, fraction of the cluster, and absolute neuron count of group/cluster intersection. Within cluster, groups are arranged in ascending order of the group fraction.

### Pairwise morphometric comparisons of neuron groups identified by cluster analysis

**Figure 6:**
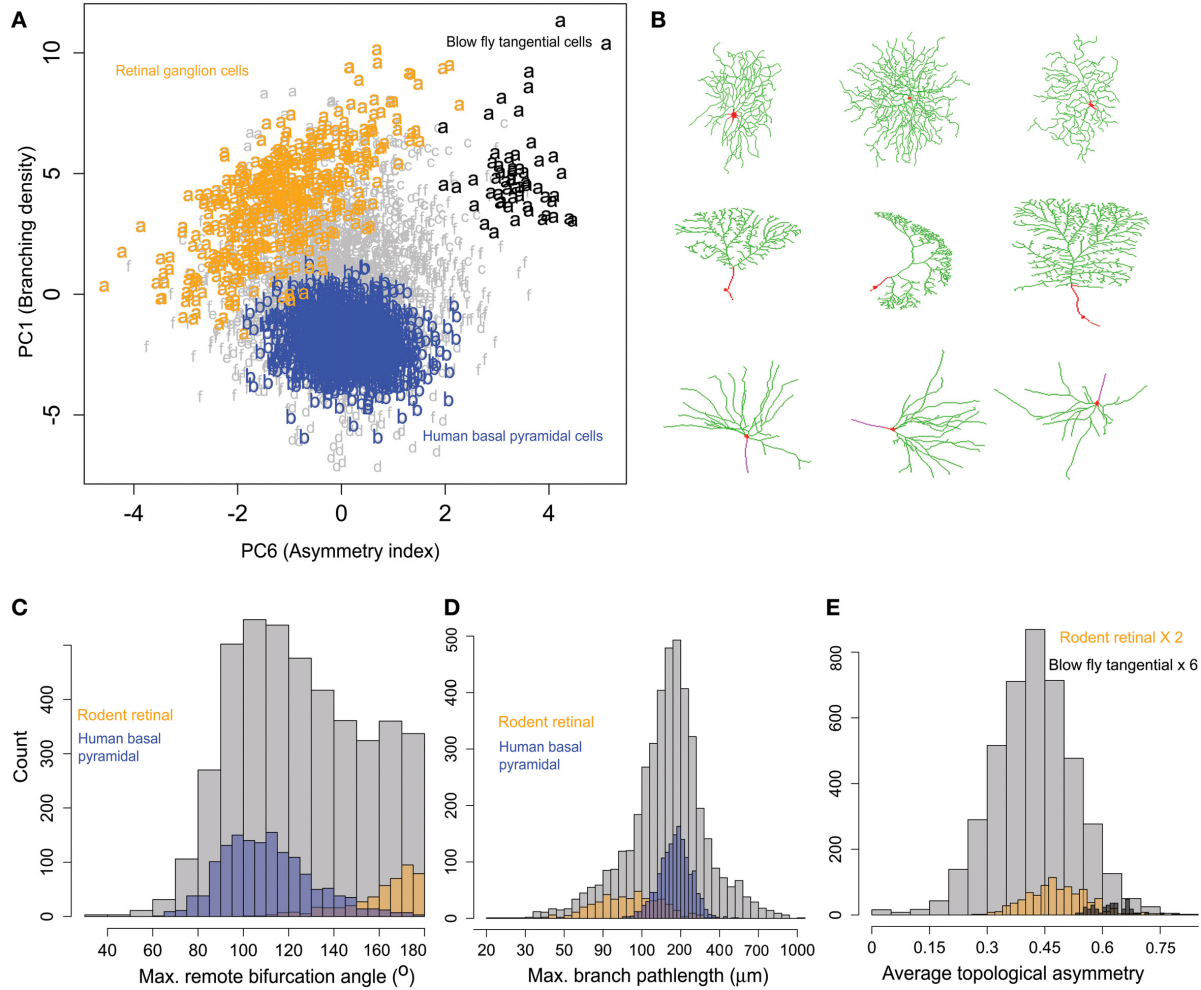
**Similarities and differences of rodent retinal ganglion cells with other neurons within and across clusters. (A)** All rodent retinal ganglion cells together with blowfly tangential cells (cluster *a*) show highest branching density along PC1 compared to others metadata groups. The human basal pyramidal cell cluster (*b*) is highlighted for comparison. PC6 separates the tangential and ganglion cells, showing sub-cluster differences. The retinal cells also show a pattern of increasing partition asymmetry with increasing branching density. **(B)** Sample images of retinal ganglion cells (top), blowfly tangential cells (middle), and human basal pyramidal cells (bottom). NeuroMorpho.Org IDs of these neurons from left to right: 06464, 05352, 05405, 06652, 01895, 06640, 03723, 03724, 03722. **(C)** Rodent ganglion cells have larger amplitude angles compared to human basal pyramidal cells (and most other cell classes). **(D)** Rodent ganglion cells also display shorter branch length, corresponding to higher branching density. **(E)** The blowfly neurons, while sharing similar branch path length and amplitude angles with the retinal cells, have higher topological asymmetry.

Exploratory inspection of neuronal clusters in the 6-dimensional space of principal morphometric components together with the association between clusters and metadata groups (Tables 3, 4) suggested closer inspection of specific morphological features in selected pairs of neuronal groups defined by their species, brain region, and cell type. The first example pertains to rodent retinal ganglion cells (Figure 6), which are characterized by high branching density and related morphological features (e.g., wide bifurcation angles). These neurons, pooled from mice and rats in four different archives, constitute 80% of cluster *a*, the farthest away from the center (Figure 5C and Table 4). At the opposite end along the first principal components is cluster *b*, entirely made of human pyramidal basal dendrites. Visual inspection (Figure 6B) reveals the distinctive shapes of rodent ganglion cells and human basal dendrites. Statistical analysis of the two main morphological loadings of PC1 (bifurcation amplitude and branch path length) confirmed the considerable difference between these two neuron groups, even when including those found in clusters other than *a* and *b* (Figure 6C).

The second most prominent group in cluster *a* is constituted by blowfly tangential sensory neurons. These neurons share with the rodent ganglion cells not only comparable branching density properties captured by PC1 (low branch path length and high bifurcation angle), but also similar distributions on PC2 through PC5 and all corresponding morphological features loading on those dimensions. These include measures of size (e.g., total dendritic length and spanned volume), of space filling (fractal dimension and tortuosity), and of arbor planarity (torque and tilt angles). Such tight alignment on the first five principal components along with the morphological co-clustering suggests a structural basis for the functional commonalities between blowfly tangential cells and retinal ganglion cells, both of which process motion-sensitive visual information (Kong et al., 2005; Cuntz et al., 2008).

Nevertheless, rotation on the sixth principal component exposed a surprising and nearly perfect separation between retinal ganglion cells and blowfly tangential cell (Figure 6A). Since the main morphological feature loading on PC6 is topological asymmetry (the average partition of terminal degree over all bifurcations), we compared the distribution of this measure between the two neuron classes (Figure 6D). This analysis demonstrated that blowfly tangential neurons have much more asymmetric bifurcations than ganglion cells (and most typical neurons). Interestingly, the data projection over the first and sixth principal components (Figure 6A) also suggested a linear relationship between topological asymmetry and branching density in rodent retinal ganglion cells but not in other groups. The Pearson correlation coefficients for branching density and asymmetry index (*R* = −0.50) and for bifurcation amplitude remote and asymmetry (*R* = 0.51) are both statistically highly significant (*p* < 10^−10^).

**Figure 7:**
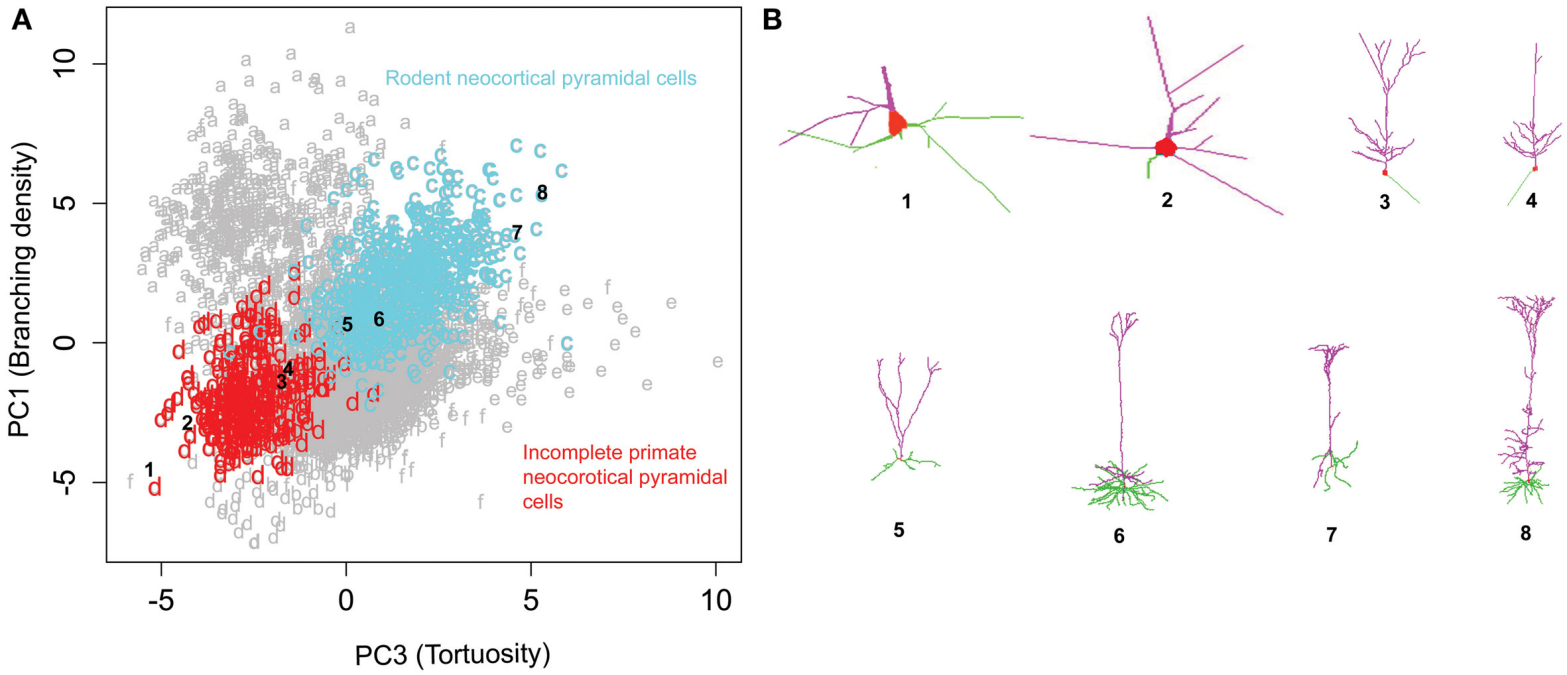
**Rodent and primate cortical pyramidal cells show a distinct linear relationship between PC1 and PC3. (A)** The majority (71%) of cluster *c* consists of rodent cortical pyramidal cells, whereas a similar proportion of cluster *d* (72%) corresponds to primate pyramidal cells, which tend to be only partially reconstructed. **(B)** Sample images of incomplete primate pyramidal cells in the top row (1–4) and rodent cortical pyramidal cells in the bottom (5–8). The numbers indicate their corresponding position in the cluster plot illustrating the progressive increase in branching density and tortuosity in both clusters. The NeuroMorpho.Org IDs of these neurons from left to right: 01821, 01526, 01627, 01623, 09630, 09474, 02569, 00266.

Rotating the data along the first and third principal components (related to branching density and tortuosity, respectively) revealed another distinct relationship across pyramidal cells from different species, brain regions, and developmental stages (Figure 7). Specifically, neocortical pyramidal cells from rodents (clusters *c*) and primates (cluster *d*) display a trend of increasing branch tortuosity with increasing branch density (Figure 7A). Visual examination of morphologies selected from the corresponding clusters in the PC1-PC3 scatter plot demonstrates a correspondence in the increase of branch density and branch tortuosity (Figure 7B). The least tortuous trees, and many of the primate neurons, are noted to be incomplete reconstructions, in which only dendrites proximal to the soma are traced. In contrast, the dendrites of rodent neocortical pyramidal neurons tend to be fully reconstructed in both apical and basal arbors.

### Critical assessment of potential confounds

In the course of the iterative process of data inspection, hypothesis formulation, research design, and quantitative analysis, we encountered numerous challenges pertaining to data validation, curation, and standardization across labs. After a preliminary exploration of the entire content of NeuroMorpho.org, we decided to include in our study only approximately half of the available neurons. Specifically, we chose to avoid multi-lab analysis of axons, because of the extreme dependence of axonal morphology on experimental conditions. In our early analysis attempt that did not segregate axons from dendrites, biological findings became practically impossible to disentangle from major artifacts. This selection effectively defines a standard of minimal requirements for effectively comparing neural arbors.

Moreover, we excluded measures related to branch diameter (branch power ratios, surface areas, occupied volume, etc.) due to their strong sensitivity on the inter-laboratory variety of labeling or staining, imaging resolution or optical magnification, and other experimental details affecting tracing conditions (Scorcioni et al., 2004). Furthermore, most reconstructed cells originate from preparations in acute brain slices (*in vitro*). In the primary somatosensory region of rat neocortex (S1), this common preparation may result in trimming off more than 50% of the dendritic arbor (Oberlaender et al., 2012). These slicing artifacts impact larger brains to a greater extent, as reflected by the fact that human cells are only represented by basal dendrites. In addition to species differences, trimming effects also depend on animal age, slicing thickness and orientation, and the depth of electrode penetration in the tissue. For these reasons, when mining the cluster analysis results, we paid particular attention to only report findings as “biological” (Figures 6, 7) that were not based on size or any morphometrics significantly affected by trimming artifacts. Instead, we identified correlations based on measures such as branching density, tortuosity, and branch angles, all of which have been previously found to be consistent between *in vitro* and *in vivo* preparations (Pyapali et al., 1998).

On the one hand, this judicious design allowed the independent reproduction of findings reported in prior publications. These included several cases of “split metadata groups” into two morphological clusters, which reflected structural differences related to the subject's gender (Anderson et al., 2009) or developmental stage (Smit-Rigter et al., 2012). On the other hand, experimental artifacts still contributed to clustering, and other splits of metadata groups between two clusters (Table 3) revealed differences likely due to staining protocol or slicing direction, which were not recognized or discussed in the original reports (Anderson et al., 1995; Soloway et al., 2002; Goldberg et al., 2003; MacLean et al., 2005; Nikolenko et al., 2007; Woodruff et al., 2009). Thus, database-wide analyses can reveal potential confounds not easily pinpointed in individual studies.

One of the most common artifacts of tissue processing is shrinkage, and this factor is also highly variable among labs. Shrinkage differentially affects the slice planar and perpendicular dimensions (the latter typically producing a larger effect). Thinner slices tend to shrink more and so do preparations from younger animals. The duration of the experimental procedure may also impact shrinkage, as do the bathing and embedding media. Shrinkage can be measured in all dimensions and it can therefore be compensated for by multiplying the resulting position coordinates by an appropriate correction factor. However, this post-processing operation also exacerbates noise due to light diffraction and other experimental errors. These sources of errors tend to be larger in the direction corresponding to the depth of the slice (“Z”), which is usually estimated through a piezo-controller in the motorized stage. Moreover, shrinkage typically varies both within and between sections, and an accurate calibration therefore requires multiple repeated measurements that add to the already demanding labor intensity of digital reconstruction. For these reasons, shrinkage is not always measured, reported or corrected for. This variability across published studies further worsens the numerous sources of differences due to experimental processing.

In light of the above consideration, we specifically looked for potential shrinkage-related confounds in the clustering results. Out of 56 unique combinations of clusters, metadata groups, and corresponding published articles, only 14 reported shrinkage estimates or mentioned shrinkage altogether. Of those, a mere 5 applied the corresponding correction to the data. Unsurprisingly given the limited sample, we found no statistically significant association between both corrected or uncorrected values and clustering. Next, we examined slicing thickness, which was reported in 49 (out of 56) cases (with median 200 μm). Values varied broadly from 80 to 400 μm, with 85% of them falling between 120 and 350 μm. No statistical association was found between clustering and these values. The lack of explicit shrinkage information prevents firm conclusions and leaves open the possibility that some of the findings we report may be ultimately due to slicing artifacts. However, the low coefficient of variation of measurements typically sensitive to shrinkage, especially tortuosity and fractal dimension (Table 1), suggests that the noise related to shrinkage (as opposed to that affecting size measures) may affect most of the analyzed data to a similar degree.

Fully assessing the potential usefulness of the reported results will require additional investigation. For example, morphologically detailed electrophysiological simulations might be useful to explore how the observed relations between datasets (Figure 6) or between morphological variables (Figure 7) could affect input/output relationship of individual neurons (e.g., Scorcioni et al., 2004; Komendantov and Ascoli, 2009). Similarly, the effect of these morphological relations on potential network connectivity could be studied by embedding the digital reconstructions in an appropriate three-dimensional model of the surrounding neural tissue (e.g., Chiang et al., 2011; Ropireddy and Ascoli, 2011). The continuous expansion of the available pool of neuronal reconstructions will also enable the future validation and refinement of these results with additional or independent datasets.

## Discussion

This work illustrates how shared morphological data can lead to new observations of potential neurobiological interest by enabling statistical quantification of commonalities and differences among neuron groups. However, our results also demonstrate the challenges of working with large-scale datasets from heterogeneous sources, even after extensive effort in metadata curation and management as well as in data standardization and selection. Direct analysis of selected morphometric features among large neuron groups organized by the main metadata dimensions of species, brain region, and cell type failed to reveal meaningful patterns beyond the well-known variability of neuronal shape. At the same time, systematic pairwise examination of all 45 neuronal groups with distinct species, brain region, cell type, and lab of origin for each of the 27 main morphological features would produce more than 50,000 comparisons, raising questions of scientific interpretation and statistical significance.

To overcome these issues, we adopted principal component analysis to identify the most discriminant morphological features throughout the dataset, and model-based cluster analysis to segregate neuron groups solely on the basis of the morphometric characteristics. This approach allowed rigorous examination of the statistical associations between clusters and metadata and inspection of the most informative morphological measurements on the basis of their principal component loadings. The results revealed morphological differences between specific cell types and animal species that were robust to lab provenance while retaining considerable sensitivity to developmental stages and fine regional location as well as to the original experimental conditions. For example, neocortical pyramidal cells from rodents and primates alike display a trend of increasing branch tortuosity with increasing branch density (Figure 7A). This distinct relationship, holding across different species, brain regions, and developmental stages, appears robust to slicing artifacts as demonstrated by the co-alignment of both partially reconstructed and fully reconstructed neurons (Figure 7B).

The primary features of dendritic morphology corresponded to branching density, size, space filling, and bifurcation asymmetry. Of these features, size is likely to be the most dramatically impacted by differential trimming artifacts from brains of varying size. Nevertheless, the most interesting biological findings were based on branch- or bifurcation-level observations. Rodent retinal ganglion cells stood out for their extreme branching density, and clustered together with other neuron types involved in primary sensory processing as well as with developing pyramidal cells from the somatosensory cortex of 6–9 day-old rat. Moreover, the results also highlighted species differences within the same cell types by differentiating retinal cells of rodent from those of fish and amphibians. Specifically, ganglion cells have denser branching and wider bifurcation angles in rodents than in non-mammalian vertebrates (Figures 5B, 6, Table 2). This observation is based on pooling of mice and rats data from four different labs in one cluster, and of fish and salamander from two different labs in the other, and we failed to find any methodological reasons that could explain these morphological differences.

Blowfly tangential sensory neurons are similar to the rodent ganglion cells in many morphological features (e.g., low branch path length, comparable fractal dimension, tortuosity, and arbor planarity), possibly providing a geometric correlate for their similar function in processing motion-sensitive visual information (Kong et al., 2005; Cuntz et al., 2008). Nevertheless, retinal ganglion cells and blowfly tangential cells can also be neatly distinguished due to the much more asymmetric bifurcations of the latter neurons (Figure 6A) relative to those of the former (and of most typical neurons). Interestingly, cluster analysis also suggested a linear relationship between topological asymmetry and branching density in rodent retinal ganglion cells but not in other groups, pointing to a previously unrecognized peculiar morphological signature of this class only.

The branching density of mature cortical pyramidal cells, in contrast, was at the opposite end relative to ganglion cells (also demonstrating the effect of developmental changes) and displayed a distinctive correlation with branch tortuosity. Adult neocortex pyramidal cells represent the largest population in NeuroMorpho.Org and come from a broad range of animals, anatomical subregions, layers, and experimental conditions, enabling certain morphological differentiations (e.g., rodent S1 vs. primate M1). Non-cortical neurons, including striatal, olfactory, and others, were distinguished for the smaller size and larger variability of their dendritic arbors.

Several recent investigations have adopted similar analysis designs and strategies for dimensionality reduction, mainly for the purpose of exploratory neuron type classification (e.g., Kong et al., 2005; McGarry et al., 2010; Santana et al., 2013). Alternative approaches to develop automated machine-learning classifiers for identifying neuron types also promise to be effective for large data sets. The present exploratory study used multivariate morphometric analysis to identify the most informative morphological features that distinguish between neuron groups organized by their metadata. We predict that statistical morphometric mining will also prove to be useful for developing quantitative hypotheses and for designing computational models of dendritic growth. At the same time, we discussed the considerable challenge of pooling together data from disparate experimental conditions, and the resulting analysis limitations.

Generation of standardized morphological data across laboratories and research designs could yield much more powerful large-scale data mining. In particular, we are convinced that better clustering would result from more consistent data collection. Systematic reliability assessment of experimental protocols can maximize morphological reproducibility and minimize tracing artifacts (e.g., Dercksen et al., 2014). Any such improvements would also help refine cluster analysis by reducing variability. Unfortunately, the arguably “ideal” experimental choices (*in vivo* labeling, reconstructions at the resolution limit of light, systematic measurement and compensation of tissue shrinkage, serial tracing across histological sections, etc.) also correspond to the most labor-intensive conditions for manual or semi-manual morphological reconstructions. This tension between quality, sample size, and research cost underscores the need and desirability of fully automated and broadly applicable tracing technologies (Brown et al., 2011; Donohue and Ascoli, 2011).

### Conflict of interest statement

The authors declare that the research was conducted in the absence of any commercial or financial relationships that could be construed as a potential conflict of interest.
